# *AirKit*: A Citizen-Sensing Toolkit for Monitoring Air Quality

**DOI:** 10.3390/s21124044

**Published:** 2021-06-11

**Authors:** Sachit Mahajan, Jennifer Gabrys, Joanne Armitage

**Affiliations:** 1 Computational Social Science (COSS), ETH Zürich, 8092 Zürich, Switzerland; sachit.mahajan@gess.ethz.ch; 2 Citizen Sense, Department of Sociology, University of Cambridge, 16 Mill Lane, Cambridge CB2 1SB, UK; 3 School of Media and Communication, University of Leeds, Woodhouse, Leeds LS2 9JT, UK; J.L.Armitage@leeds.ac.uk

**Keywords:** low-cost sensors and devices, sensor design, crowd sensing and crowd sourcing, social networks, smart cities, social impacts, air pollution

## Abstract

Increasing urbanisation and a better understanding of the negative health effects of air pollution have accelerated the use of Internet of Things (IoT)-based air quality sensors. Low-cost and low-power sensors are now readily available and commonly deployed by individuals and community groups. However, there are a wide range of such IoT devices in circulation that differently focus on problems of sensor validation, data reliability, or accessibility. In this paper, we present AirKit, which was developed as an integrated and open source “*social IoT technology*”. AirKit enables a comprehensive approach to citizen-sensing air quality through several integrated components: (1) the Dustbox 2.0, a particulate matter sensor; (2) Airsift, a data analysis platform; (3) a reliable and automatic remote firmware update system; (4) a “Data Stories” method and tool for communicating citizen data; and (5) an AirKit logbook that provides a guide for designing and running air quality projects, along with instructions for building and using AirKit components. Developed as a social technology toolkit to foster open processes of research co-creation and environmental action, Airkit has the potential to generate expanded engagements with IoT and air quality by improving the accuracy, legibility and use of sensors, data analysis and data communication.

## 1. Introduction

Cities worldwide face numerous challenges related to socio-economic and environmental sustainability and justice [1]. Air pollution is one such challenge that adversely impacts human health [2,3,4]. Air pollution is one of the leading causes of death worldwide, with pollution often concentrated in urban areas. Air pollution is a leading risk factor for non-communicable diseases and accounts for 22% of all deaths from cardiovascular-related disease, 26% of deaths related to ischaemic heart disease, 25% of deaths related to stroke, 53% of deaths related to chronic obstructive pulmonary disease and 40% of deaths related to lung cancer [5]. Air pollution can also contribute to climate change, and an increase in greenhouse gases can lead to extreme weather conditions such as heat waves that can have further negative impacts on health and environments.

In this context, Internet of Things (IoT) technologies have been used to work toward more inclusive, sustainable and resilient cities by documenting pollution hotspots. Conventional methods for air quality monitoring by regulatory bodies involve using fixed stations that are highly accurate but expensive. Because these monitors are located in a limited number of locations, it can be difficult to achieve a spatially dense or extensive monitoring network [6]. IoT approaches to monitoring air quality can provide data across more expansive and nuanced locations, thereby revealing temporal and spatial patterns of pollution that might have been overlooked. The application of low-cost IoT sensor nodes has already changed the paradigm of air quality monitoring. Such low-cost sensing solutions have the potential to supplement regular air quality monitoring networks, enable personal exposure monitoring and promote environmental engagements by providing digital assistance [7]. This IoT approach can provide greater coverage at a reduced cost while shaping policy and decision making for urban areas that might not be adequately monitored by existing regulatory infrastructures.

**Figure 1:**
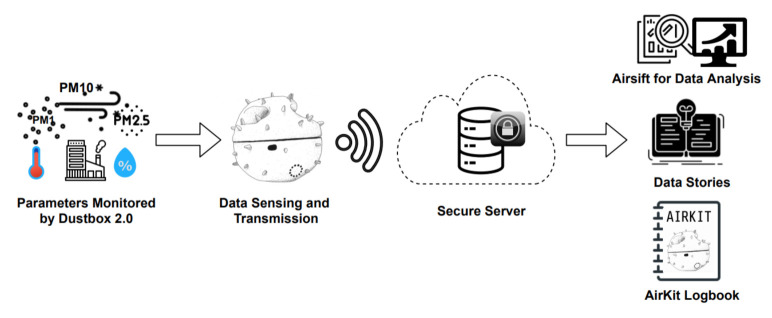
Overview of AirKit framework.

The AirKit framework presented in this publication builds on ongoing Citizen Sense research into air quality monitoring, including through the use of a first-generation Dustbox [8]. Citizen Sense is an ERC-funded research project that investigates the rise in environmental-sensing technologies, which asks how or whether these devices enable expanded forms of citizenship. AirKit received additional ERC proof of concept funding to develop a comprehensive citizen-sensing infrastructure that would consolidate and build upon technologies and practices developed throughout the Citizen Sense research. Figure 1 gives an overview of this AirKit framework.

AirKit is especially novel because it emphasises how the social aspects of IoT are a crucial component of technological innovation [9]. In contrast, most sensors focus on the technical configurations of sensor hardware and data collection, which can be more or less open. This proof-of-concept project includes a Logbook with instructions for setting up air quality campaigns; a second-generation Dustbox 2.0 with several unique features, including over-the-air programming, a redesigned 3D-printed enclosure, and instructions for DIY calibration; an Airsift citizen-led data analysis platform; and a Data Stories tool for communicating data and proposing air quality actions and interventions. AirKit then consists of more than sensors and data. It is an end-to-end citizen infrastructure for monitoring air quality, with components that are openly available for widespread use and further development.

While the availability of low-cost sensors has significantly increased options for conducting more pervasive environmental monitoring, data accuracy still remains a significant challenge [10,11,12]. Over the years, studies have found the importance of performing pre- and post-deployment colocation with reference monitors [13,14] for improving the accuracy of low-cost air quality sensors. However, one of the questions that remains unanswered is how to create trust across communities, scientists and regulatory bodies. We answer this question by focusing on data reliability and transparency, which are achieved by performing extensive sensor validation and evaluation in real-world scenarios in collaboration with the regulatory bodies.

Another emerging issue related to IoT devices is scalability [15]. With growing numbers of connected IoT devices playing an increasingly critical role in infrastructure, it becomes important to design systems that can regularly monitor software and provide secure and reliable updates. Most of the available IoT devices lack a built-in and secure automatic update system [16]. In a scenario where there is a deployment of thousands of IoT devices, it is not possible to check every device manually and update it in case of a software glitch or firmware update. There are some basic over-the-air (OTA) firmware update methods available, however, the disadvantage of such systems is that they need the device and the update server to be connected to the same local network and they do not have a standard manifest that is needed for a secure update [16]. Most of the existing firmware update methods are hardware and operating system specific [17,18], and the ones used by existing IoT devices [6,19] require the user to perform the update manually. We address this issue by proposing an automatic remote update method that encompasses all steps of the update process: from firmware generation and validation, to transmitting the update to the IoT device, its verification and installation.

**Figure 2:**
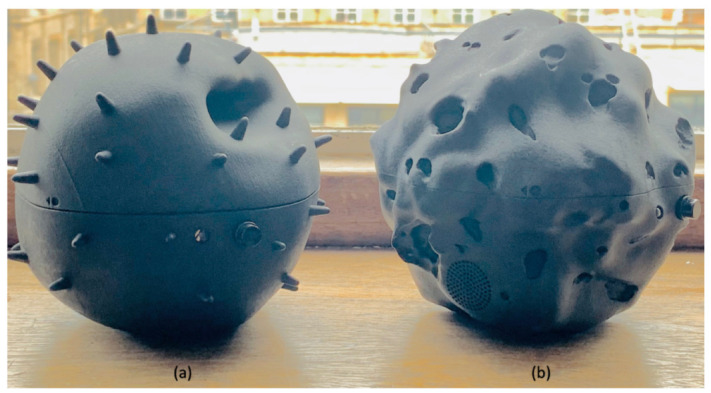
Dustbox 2.0 shapes: (**a**) pollen particle and (**b**) diesel char particle.

With newer low-cost sensors and more economical 3D-printing techniques, it has become easier than ever to assemble a low-cost sensor device in a cost-effective manner. However, an additional challenge that arises is how to develop an enclosure design to enable the IoT framework [20]. A robust and secure enclosure design not only improves the overall performance of the device, but also shields the components from potentially harmful ambient conditions [21]. We address these challenges in two phases. During the enclosure design phase, we created a modular and future-proof design that is weatherproof and resistant to adverse weather conditions. The top shell of the Dustbox 2.0 enclosure is designed with small channels that prevent water from pooling on the surface or leaking inside the device. As shown in Figure 2, the Dustbox 2.0 consists of two different particulate-matter shapes when viewed under an electron microscope: the first is based on the form of pollen particles, and the second is based on the form of diesel char particles. During the evaluation phase, the performance of the Dustbox 2.0 is assessed in a real-world environment.

The key contributions of this paper can be summarised as:

1. AirKit is presented as an end-to-end citizen-sensing infrastructure for air quality monitoring. It is designed as an integrated IoT framework that is based on open technology principles, including open architecture, open hardware, open software and open data.
2. We introduce an automatic remote firmware update mechanism that is vital for implementing new functionalities as well as resolving vulnerabilities. The mechanism allows the Dustbox 2.0 devices to securely download a verified firmware update whenever a new version is uploaded to the server.
3. We demonstrate that the Dustbox 2.0 can provide consistent and accurate air quality data, and through colocation, show that it has a high correlation with industry-grade instrumentation as well as a regulatory air quality monitoring network.
4. We highlight the potential role of IoT as a social technology that can support deeper understanding of and action on air quality issues through expanded knowledge practices of co-creation, sharing and analysis.

The remainder of this paper is organised as follows. Section 2 provides a review of related works on IoT-based frameworks for air quality sensing. In Section 3, we describe the system architecture and discuss its components in detail; in Section 4, we discuss the system implementation and results. In Section 5, we describe the evaluation process and different methods used for the evaluation of AirKit framework. Section 6 contains some concluding remarks.

## 2. Literature Review

With the advancement in information and communication technology and the availability of low-cost sensors, several low-cost air quality monitoring systems have been developed for monitoring air pollutants [22]. These systems focus on monitoring different pollutants, including particulate matter (PM) [6,19,23], carbon monoxide (CO) [24,25] and nitrogen dioxide ($NO_{2}$) [26,27]. An important aspect of creating an effective IoT air quality monitoring framework is to have an accurate system that is open and scalable. While great attention is often paid to the development of hardware and analysis techniques, we propose that technological features are just a part of setting up an accurate low-cost sensor network. Here, we draw attention to the documentation and instructional details of projects and how they describe the build, calibration, installation and maintenance of sensors, as well as the interpretation of data. Within this context we also consider the motivations and goals of different sensing projects and how they shape project design and materials. In understanding and actioning data, we consider the integration of different datasets and how they are represented, analysed and used by citizens. In this section, we look at recent open source participatory air quality monitoring projects to analyse key concerns that informed the development of the AirKit toolkit. We focus on projects that monitor PM2.5, where much development has taken place and sensors are more reliable. In our analysis, we consider how different projects produce sensors, data visualisation tools, instructions and documentation. We thoroughly reviewed the existing IoT-based air quality monitoring frameworks and broadly classified them into six categories based on the fundamental building blocks of an IoT system:

**Motivation and Goals**: Sensor toolkits are shaped by project motivations. These projects are led by different entities including academic institutions and maker-spaces as well as collaborations between the two. The majority of projects cite central aims of developing sensors to facilitate citizen engagement (i.e., Smart Citizen Kit (SCK) [19]), development of decision-making tools (i.e., AirBox [28]) and to increase citizen awareness of air quality issues (i.e., hackAir [29]). In all of these projects, citizens are encouraged to participate in air quality monitoring by building a sensor or setting up an existing sensor. Awareness of air quality issues and citizen participation in monitoring is important, not least because of the benefits of low-cost high-density monitoring. We propose that an effective toolkit should also be concerned with how these data can be analysed and developed into narratives by citizens to intervene in local air quality policy. Existing sensors have been used in these activities where local authorities and organisations have developed guidelines and methods for using monitors to inform clean air policies (i.e., PurpleAir [30]).

**Hardware Components**: IoT sensor projects include a range of electronic hardware, including particulate matter and temperature and humidity sensors, micro-controllers with an additional or integrated wireless chip. Additional parts include LEDs for indicating sensor status and other peripherals such as buttons (SCK [19], AirBeam2 [31]). A number of low-cost sensors are available for measuring PM2.5. For those using the Nova PM SDS011, PM10 can also be measured (hackAir [29] and Luftdaten [32]) and the Plantower PMS5003 range produces readings for PM1, PM2.5 and PM10 (AirBox [6]). These sensors have been found to offer suitable accuracy and good correlation with industry-grade instruments. These sensors are calibrated in the lab and send values in μg/m${}^{3}$ to the micro-controller through the serial port. The projects use temperature and humidity sensors including the BME280 (AirBox [6]) and DHT22 (hackAir [29]). Many projects use micro-controllers that are integrated with the ESP8266 Wi-Fi module including Node MCU (Luftdaten [32] and Wemos D1 Mini (hackAir [29]) or build custom boards (SCK [19]). Micro-controller units such as ESP32 and ESP8266 have been widely used for air quality monitoring applications [33,34]. These units are highly durable and compact and provide cost-effective and integrated solutions for IoT applications. Other sensors use the MediaTek 7688 (Airbox [6]). Devices often implement the low-cost open source prototyping environment Arduino IDE due to the availability or libraries and usage by electronics hobbyists and makers. Many devices do not employ peripherals, such as LEDs and buttons, which can reduce the cost and complexity of designs (hackAir [29], Luftdaten [32]). However, this can have some impact on usability in terms of monitoring the status of and debugging the sensor. The state of the art in these sensors is constantly evolving. Here, the importance of open source technology comes to the fore, where devices can be re-built and improved once projects are no longer sustained by their initiators.

**Firmware**: The firmware on devices should be updated in accordance with security vulnerabilities, changes in protocols and fixing errors. Devices can be programmed using a USB connection or through remote protocols. SCK [19] firmware updates require a USB connection through the Platform IO software. The Airbox [6] uses a protocol where updates are released on GitHub and users have to manually update the device if a new firmware version is available. hackAir [29] have developed a library for their sensor which requires citizens to install Arduino and the hackAIR library (amongst others) and upload it to the micro-controller via a USB connection. Updating firmware over physical USB connections and manual updates requires some knowledge and experience of the hardware and platforms. We suggest that remote, automated updates offer greater security for sensors and lesser technical expertise.

**Cost**: The total cost of each sensor in these projects normally accounts for the electronic hardware used such as sensors, micro-controllers and wires (i.e., hackAIR). The sensors below range from approximately EUR 200 (AirBeam2 [31] and PurpleAir [30]), EUR 90 for the SCK [19] (without an enclosure) to EUR 30 (hackAir [29]). Developing suitable enclosures for air quality monitoring devices is important in relation to protecting the internal electronics, allowing suitable airflow and enabling the device to be positioned in such a way that it produces the most accurate readings (i.e., mounting). Whilst some projects offer low-cost DIY solutions to this (such as Luftdaten), they are not necessarily reproducible across different geographic regions. The Luftdaten solution means that parts are loose within some plastic piping, making connections more vulnerable to sharp movements and humidity. Devices that are used outdoors should be sealed and waterproofed with suitable airflow to and from the sensor inlet/outlet. PurpleAir has achieved this by placing devices within a sealed unit with an open bottom. While this allows the flow of air and access to a USB port for powering, the device is difficult to disassemble. Mounting solutions across the devices vary and are often insufficiently documented. Sensors should be mounted so that they are secure from high winds, at a suitable height, away from objects that might obscure or distort readings (i.e., boiler outlets) and protected from moisture. At the same time, they should be close to a wireless network and power source. The installation of devices can be challenging as it is dependent upon available infrastructure and normally requires a solution to suspend the sensor. While rope and string can be used, these solutions are not particularly secure in the long term.

**Data Visualisation and Analysis**: These sensors pipe data to a web platform where particle concentration is represented through colour or numerical values across a geographical space (Luftdaten [32]). Some sensors offer a basic graphing capability that indicates peaks in particle concentration within a time period. Others have the option of downloading datasets. Airbox [6] includes data-based services for forecasting [35], visualisation, anomaly detection [36] and a healthy route recommendation application [37]. Here, advanced plots such as those provided in the Open Air library (http://davidcarslaw.github.io/openair/) (accessed on 10 November 2020) allow a more in-depth understanding of pollution sources and how weather affects air quality.

**DIY Building Instructions**: The participatory and open source nature of these projects asks citizens to engage in the production of sensors. This means that projects have accompanying websites where sensors are documented (AirBeam2 [31]) or more explicit instructions are given as text, image and video (SCK [19], Luftdaten [32], CanAirIO Sensor [38] and hackAir [29]). These instructions often focus exclusively on building sensors (hackAir [29]). The SCK offers instructions for deploying sensors focused on mounting techniques, as well as advice on calibration techniques and an overview of a data analysis framework. Generally, the documentation for each project reflects its central aims—to engage and educate citizens on air quality. With these projects, we see that the production of sensors and data is prioritised. We suggest that a toolkit must also include documentation to guide citizens in establishing monitoring projects; installing, maintaining, calibrating and positioning sensors; health and safety guidance; analysis techniques; as well as methods for narrating data and developing action points.

With the availability of integrated low-cost air environment monitoring solutions, it is clear that the technology for real-time citizen sensing is advancing rapidly. However, such solutions have some major limitations, including project setup, validation [39], data reliability [40], security [16] and accessible tools for data analysis. AirKit aims to create an accurate, open and reliable air quality monitoring system, while investigating the role of low-cost sensors and digital monitoring technologies in facilitating and organising new types of environmental engagement.

## 3. System Architecture

**Figure 3:**
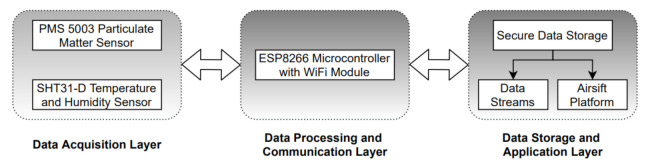
System architecture.

The AirKit framework is based on open hardware, open source software and open data. Figure 3 shows the system architecture of the framework, which comprises three major components:

- *Data Acquisition Layer*: The sensing layer is the foundation of the AirKit system. Dustboxes are the sensing units and are the main entities of this layer that sense and report particulate-matter, temperature and humidity data. The monitoring nodes are suitable for indoor and outdoor use. We tested two variants of the Dustbox, however, both have similar internal components and wiring. One of the key elements of the Dustbox device is that they are completely open source. The hardware and software details are openly available so that people can build their own devices as well as examine them for data quality. The details of the Dustbox hardware and software are provided in the next section.
- *Data Processing and Communication Layer*: The data processing and communication layer is responsible for processing and integrating different sensor data and transmitting it to the data storage and application layer. A WLAN network based on the IEEE 802.11 set of standards is used to provide a ubiquitous connection between the sensors and the Access Point (AP). Based on the existing literature, we compared multiple protocols and chose the Hyper Text Transfer Protocol (HTTP) because of the existing infrastructure and high transmission reliability [41], however, this also means a trade-off between reliability and power consumption.
- *Data Storage and Application Layer*: This layer is responsible for data storage as well as providing interactive visualisations and services to participants. The tasks can be divided into two parts. At first, data received from the Dustboxes are stored in the database. Post-processing functions are applied to the data if needed, for example, using a calibration factor or data aggregation. The next task includes hosting a server to show the data on the Airsift platform. Airsift is a web-based tool that can be used to explore, analyse and compare Dustbox 2.0 data. Different graphs and interactive features enable the real-time and historic visualisation of sensor data.

## 4. System Implementation and Results

### 4.1. Hardware Implementation

The Dustbox 2.0 is a citizen-sensing device designed by Citizen Sense to undertake environmental monitoring. It has been designed based on our previous experience of sensing air quality with the Dustbox 1.0 in Southeast London from 2016 to 2018. The Dustbox 2.0 measures three variables: (1) particulate matter in the air; (2) temperature; and (3) humidity. The Dustbox 2.0 is easy to install and can be integrated with existing urban infrastructure such as lampposts and traffic signal poles.

When designing a citizen-sensing monitor, it is important to select the most suitable sensors. Low-cost sensors have been widely used in many air quality sensing projects. However, a thorough and systematic evaluation of existing sensors is needed to select the best combination for a particular scenario. The Dustbox 2.0 uses a Plantower PMS5003 particulate matter sensor. The PMS5003 was selected based on existing sensor benchmarking studies [12,42,43,44] that show a high correlation of Plantower sensors with calibration monitors, low cost (GBP 15.00) and the overall size of the unit. The sensor is readily available at a number of DIY-electronics retailers. These sensors have laser counters to measure airborne particulate matter. There is a small fan that draws air through the sensor and passes it through a laser that can detect the concentration and size of particles in the air. The Dustbox 2.0 measures particulate matter, temperature and humidity every minute, however, the sampling time can be changed based on different monitoring scenarios. Having such fine-grained data can help in observing and understanding even small variations in the surrounding air. These data can be viewed on the Airsift (https://airsift.citizensense.net/) (accessed on 11 November 2020) data analysis tool.

**Figure 4:**
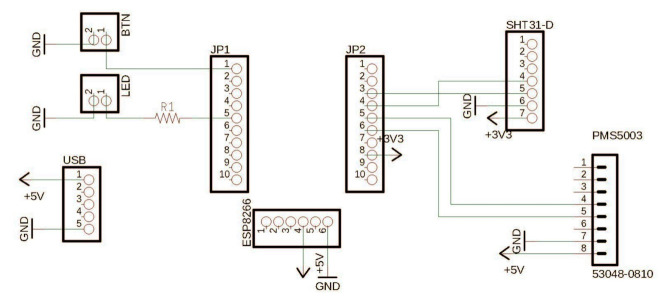
Dustbox 2.0 schematics.

**Figure 5:**
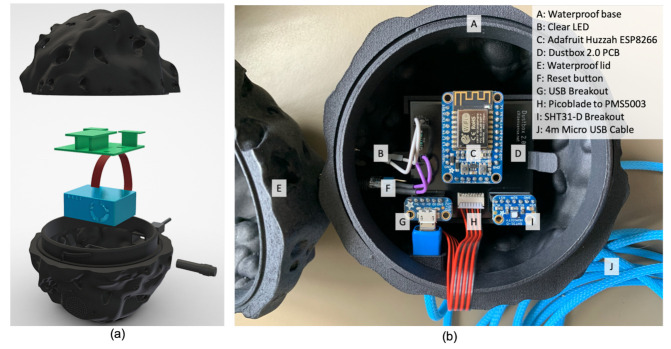
Dustbox 2.0: (**a**) 3D exploded view; and (**b**) exploded view with annotations.

The hardware for the Dustbox 2.0 includes a PCB to which the sensors, micro-controller and peripherals (LED and button) are connected. Figure 4 shows the schematics of the Dustbox 2.0. Figure 5 shows the 3D exploded view of the Dustbox 2.0 with components and annotations. These components are housed within the 3D-printed enclosure and powered using a 4 ft USB cable to provide a 5 V supply. We calculated the power consumption of the Dustbox to be in the range of 0.5–2 kWh for one month. This range is based on the power usage of all electronic components oscillating between active and standby states. The PCB is mounted with headers that allow parts to be clipped into place. For the micro-controller, we used the Adafruit Huzzah ESP8266 breakout board, which was selected due to its compact form, adequate number of inputs and outputs and embedded ESP8266 chip. This was connected to the PMS5003 sensor using a Molex Picoblade connector and a cable through which serial data are received. A Sensirion SHT31-D temperature and humidity sensor was connected to the micro-controller via digital pins. To select the temperature and humidity sensor, we compared a range of commonly available models including the BME680 and DHT22. We found that the SHT31-D offered a suitable resolution of readings (${}^{\circ }$Celsius to two decimal places) and cost (approximately GBP 8.22). We found that when compared, the BME680 offered readings approximately 2 ${}^{\circ }$C higher than the other two sensors.

There is a button connected to the reset pin of the ESP8266 to allow the device to be reset without opening the enclosure. This is connected via standard prototyping jumper pins that are soldered around the loops of the button. A clear LED, attached via jumper cables, is connected to the ESP8266 and flashes when the device is powered on and indicates whether the device is connected to a network. These connections allow for the device to be easily assembled and disassembled. The device is powered by a 5V USB supply, which is connected to a micro USB breakout board housed inside the enclosure.

The enclosure for the Dustbox 2.0 is 3D-printed in Multi Jet Fusion (https://www.materialise.com/en/manufacturing/3d-printing-technology/multi-jet-fusion) (accessed on 11 January 2021) PA 12. It is made up of two halves that slide and clip into place. All electronic components sit in the bottom half of the housing, and the top half acts as a lid. The PMS5003 sensor clips into the bottom of the enclosure and the fan faces a mesh of holes to allow airflow. The PCB sits above and not in direct contact with the PMS5003 as the sensor casing is grounded. This is kept in place with two clips. The button is inserted into the enclosure from the outside and then connected to the PCB. The LED twists and locks into place in the enclosure. The USB is inserted through a hole designed to be compatible with the Pexon series of cables. The cables are 4 m long, which allows a good distance for the sensor to be placed outside. This can be extended using waterproof cable extenders. The approximate weight of the Dustbox is 300 g. The approximate cost of a self-built Dustbox unit is GBP 200. At the time of writing, the Dustboxes can be built by following instructions available in the AirKit Logbook, or by contacting Citizen Sense to discuss a sensor deployment. Plans for commercial development are in the exploration stage.

**Figure 6:**
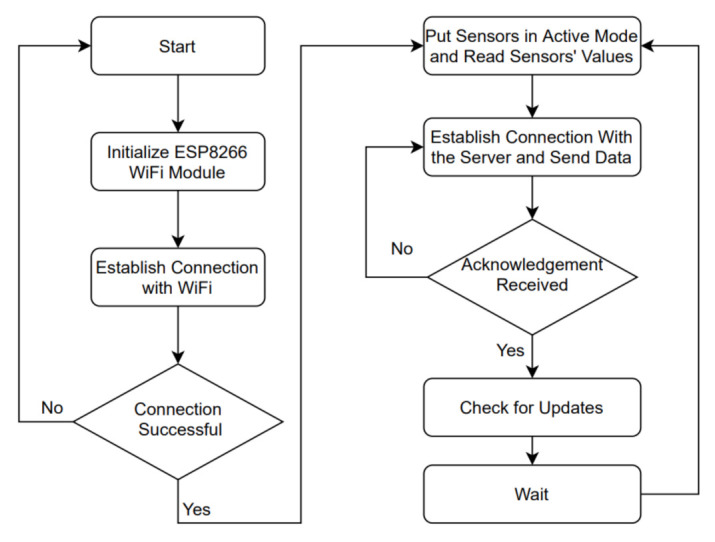
Flowchart of Dustbox 2.0 firmware.

Figure 6 shows the flowchart of Dustbox 2.0 firmware. The Dustbox 2.0 firmware performs three main tasks: (1) establishing network communication and managing related tasks; (2) simultaneously using multiple sensors; and (3) performing OTA firmware updates.

### 4.2. Dustbox 2.0 Firmware

Dustbox 2.0 uses an ESP8266 chip to talk to the particle sensor and provides all the basic network-related tasks, including creating an AP, connecting to a Wi-Fi network, obtaining date and time via an NTP protocol and uploading data to the Citizen Sense server. This ESP8266 chip runs a firmware developed using Arduino IDE. The first firmware upload has to be done using a serial cable and subsequently it can be done either serially or using the OTA functions. The firmware file includes all the functions to sense the particulate matter, temperature and humidity data, and send data to the Citizen Sense server over a secure transmission channel. Once the firmware is flashed successfully, the Dustbox 2.0 automatically goes into a set-up mode with an intermittently flashing LED. The ESP8266 Wi-Fi module then goes into the AP mode and lets the user connect to the temporary device-based Wi-Fi network named as “ESPXXXXXX”. After connecting to this network, participants are redirected to a web interface where they are prompted to provide their Wi-Fi credentials. The Dustbox 2.0 will retain the Wi-Fi credentials. In the event that the Dustbox 2.0 is restarted, it will automatically connect to the saved network. Once the network is established, the firmware puts the sensors in active mode and keeps them awake for 25 s to do the measurements and send the data to the Citizen Sense server. When the device is awake, an HTTP connection is set up and the data are sent in the form of a GET request. Once the response is received from the server, the connection is closed and the device goes back to sleep. The data are posted using GET and POST requests that are based on public and private keys provided by the Citizen Sense platform when creating a data stream. The sensed data can be viewed on the Airsift data platform. The data are sensed every one minute. The firmware also controls the reset button and the LED that are used to manage user interaction with the Dustbox 2.0.

*Dustbox 2.0 OTA Update System*: Dustbox 2.0 firmware also has remote update features, meaning we can modify and update the software and the device will download the new version and update itself without needing a web interface or a manual update process. The Citizen Sense server holds the current firmware version as well as any firmware update (if available). This is a key feature of the AirKit framework, as most of the existing frameworks can only update the devices that are connected to the same network. With the remote-programming feature, we can update the firmware remotely without accessing the device or device network.

**Figure 7:**
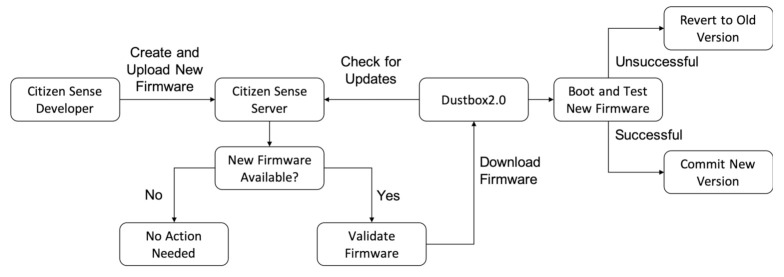
Dustbox 2.0 OTA firmware update flowchart.

*Standard OTA update library and its limitations:* The standard OTA library provided by the ESP8266 community allows the developers to perform OTA updates rather than undertaking this task through the serial port. The library uses the ESP8266 chip as an HTTP server for accepting firmware files using the HTTP POST request. Because of limited available memory, the firmware updates should be processed in fragments. The issue with the standard OTA update library is that it only uses local MD5 validation as a security protocol, which is weak [45], as the firmware binary and MD5 associated with it can be easily changed, which can compromise the device. Furthermore, while updating the firmware, there is no external verification to check the update version. To solve these issues, we modified the existing library and added the updated OTA functions to the main firmware to perform automatic and verified updates. The method is described in Figure 7.

**Figure 8:**
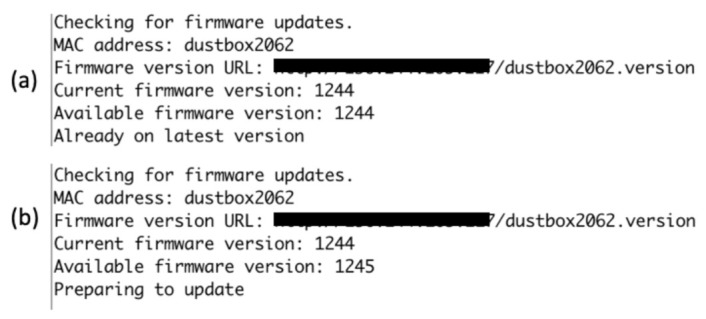
Examples of OTA functions: (**a**) no update available; and (**b**) firmware update is available.

If there is any firmware update, the Citizen Sense team prepares it and uploads it to the secure Citizen Sense server. The first step of looking for firmware updates involves verifying the identity of the Dustbox 2.0. In addition to the public and private keys, an alias name is given to every Dustbox 2.0 that needs to be verified with the server first. Once the Dustbox 2.0’s identity is authenticated, the firmware version is checked. If a new version is available, it is fetched using a GET request. The new firmware is downloaded and tested. If the process is successful, the new version is committed. If the update process is unsuccessful, the Dustbox 2.0 will revert back to the old firmware version. This is an important feature of the Dustbox 2.0 OTA system, as it makes sure that the device does not fail if the OTA image fails to boot successfully. An example of how the Dustbox 2.0 OTA system works is illustrated in Figure 8. Benefits of our OTA approach are:

- Incremental OTA updates can help in improving and updating the devices even if they are located remotely.
- Having such a system can improve scalability by increasing functionality and adding new features using regular updates.
- It is a cost-effective method as the updates are performed remotely.

### 4.3. Dustbox 2.0 Validation

In this section, we describe the validation methods that were used to evaluate the accuracy of the Dustbox 2.0. We followed three setups for validating the Dustbox 2.0: inter-unit variability, outdoor colocation and indoor colocation. Five Dustboxes were collocated with a TSI AM520 instrument. TSI AM520 is an industry-grade instrument which can monitor PM1, PM2.5, PM5 and PM10. The assessment of sensor performance was done by the determination of Pearson correlation coefficient (R) [12]. Table 1 presents further specifications and characteristics of the sensors.

#### 4.3.1. Inter-Unit Variability

We used inter-unit variability as a metric to measure the similarity between the PM2.5 data produced by different Dustbox 2.0 units. Table 2 shows summary statistics for the PM2.5 data of five Dustboxes sampled every one minute. It can be observed that all the Dustboxes show similar statistics and there is no substantial variation. The data show high correlation with an average R of 0.91. For data averaged every 1 h, the R values increased to 0.98. This shows strong linearity over the entire range of data.

#### 4.3.2. Outdoor Colocation

**Figure 9:**
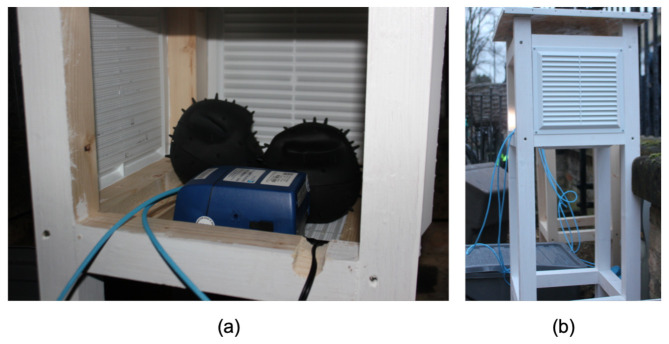
Outdoor colocation setup: (**a**) dustboxes with a TSI AM520; and (**b**) Stevenson screen setup.

**Figure 10:**
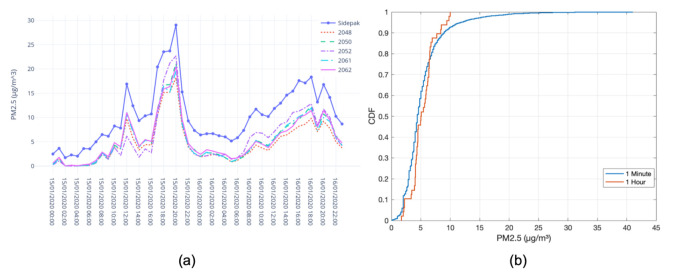
Performance comparison of Dustbox 2.0 and TSI AM520 for outdoor colocation: (**a**) line plot for hly data; and (**b**) CDF plot to understand the difference between the values recorded by Dustboxes and TSI AM520 monitor.

Five Dustbox 2.0 devices were colocated along with a SidePak TSI AM 520 instrument to enable the comparison of the Dustbox sensor output with a regulatory instrument. The site for the colocation study was strategically chosen so that the devices could be evaluated under varying environmental conditions with variable pollution levels. The colocation site was located at the city-centre right next to a busy intersection and a traffic signal, experiencing regular traffic flow and varying pollution levels during peak and off-peak hs. Figure 9 shows the outdoor colocation setup using a Stevenson screen. The aim was to evaluate the accuracy of the sensors (specifically focusing on PM2.5) and ensure that the sensors could capture sudden variations in the pollution concentration due to traffic and other emissions sources. The colocation process forms an important part of sensor quality control and data quality assurance for Dustbox 2.0. The experiment lasted for 48 h (from 00:00 on 15 January 2020 to 00:00 on 17 January 2020). The data were sampled at a rate of one sample per minute for each device. Table 3 shows the linear regression parameters and R based on the PM2.5 measurements from Dustboxes and TSI AM520 at 1 min and 1 h time intervals. The values indicate that different Dustbox 2.0 units generally had similar intercept and slope values on the same timescale. On average, the R value was around 0.83 when the samples were collected every minute, whereas the R value was around 0.98 for 1 h average PM2.5 concentration. The results show a strong linear relationship between the PM2.5 values sensed by the Dustboxes and TSI AM520. Figure 10a compares the hly data from Dustboxes and the TSI AM520 monitor. It can be observed that the Dustbox measurements follow the trends in PM2.5 concentration and are responsive to PM2.5 variations. Figure 10b compares the cumulative distribution function (CDF) of the accuracy of the Dustboxes where the x axis is the absolute value of the measurement offset between TSI AM520 and five Dustboxes. It can be observed that for 1 min sampling as well as for 1 h average, 80% of the observations have an offset below 6 μg/m${}^{3}$.

#### 4.3.3. Indoor Colocation

**Figure 11:**
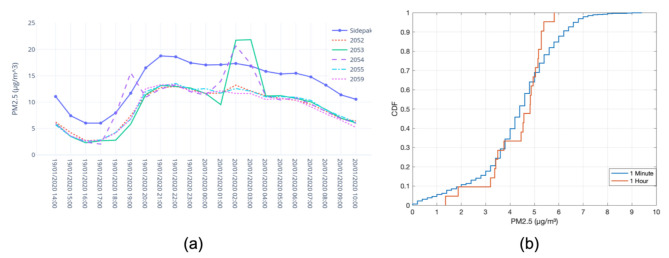
Performance comparison of Dustox2.0 and TSI AM520 for indoor colocation: (**a**) line plot for hly data; and (**b**) CDF plot to understand the difference between the values recorded by Dustboxes and TSI AM520 monitor.

Indoor colocation of the Dustbox 2.0 was performed using a TSI AM520 as the ground truth. Five Dustboxes and a TSI AM520 were colocated in a room with windows open for air circulation. The experiment lasted for 20 h (from 14:30 on 19 January 2020 to 10:30 on 20 January 2020). The data were sampled at a rate of one sample per minute for each device. Table 4 shows the linear regression parameters and R based on the PM2.5 measurements from Dustboxes and TSI AM520 at 1 min and 1 h time intervals. Overall, different Dustbox 2.0 units had similar intercept and slope values on the same timescale. There was some variation observed for Dustbox 2053 and Dustbox 2054. This was due to the outliers observed in the data of two Dustbox units. On average, the R value when the samples are collected every minute is around 0.80, whereas for the hly average PM2.5 concentration, the R value is around 0.92. The results show that there is high correlation between the PM2.5 data sensed by Dustboxes and TSI AM520 in an indoor environment. Figure 11a compares the hly data from the Dustboxes and the TSI AM520 monitor. It can be observed that the Dustbox 2.0 PM2.5 measurements show trends similar to the data recorded by TSI AM520. The time period between the 20 January 2020 at 01:00 and 20 January 2020 at 04:00 shows some outliers for Dustbox 2053 and 2054. Figure 11b compares the CDF of the accuracy of the Dustboxes where the x axis is the absolute value of the measurement offset between TSI AM520 and five Dustboxes. It can be observed that for 1 min sampling as well as for the 1 h average, almost 90% of the observations have an offset below 6 μg/m${}^{3}$.

### 4.4. Data Platform

**Figure 12:**
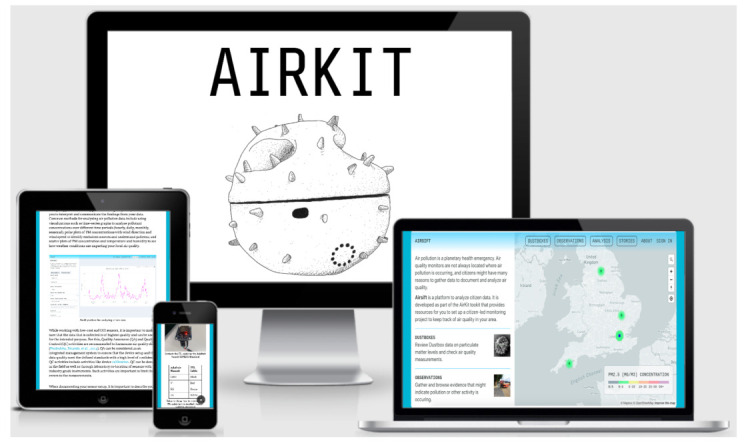
Airsift data platform and AirKit logbook.

In order to facilitate, enhance and encourage Dustbox 2.0 data access, we developed several tools and applications for data visualisation and analysis. The data platform has a responsive design and can be easily accessed via different devices, as shown in Figure 12.

*Open Data*: A data archive was setup that stores all the Dustbox 2.0 data. All the data from the Dustboxes is open and can be accessed by anyone using the Airsift platform. The data can be downloaded and imported as a CSV file using Airsift tool. This service ensures that participants can easily access the data and use it for creating data stories. It can also facilitate innovation and data-based application development. The data stories are an important element of AirKit. They provide context and meaning to the air quality data, where participants can add observations, propose actions, and publish data in formats that can be circulated to policymakers and other concerned citizens. A typical data story [46] includes compiling the data and build a compelling story that is easily understood by a wide range of audiences and key stakeholders. To facilitate openness and engagement, the AirKit Logbook [47] provides instructions for building the Dustbox 2.0 from scratch, along with instructions for setting up Dustboxes and undertaking data analysis using the Airsift platform. The Logbook also provides information about how to design and run air quality studies, and refers readers to local and regional air quality resources.

*Data visualisation and analysis*: As part of its end-to-end citizen infrastructure, the AirKit includes a DIY data analysis platform, Airsift. Airsift is web-based tool that can be used to spatially and temporally explore and analyse Dustbox 2.0 data, compare it to other Dustboxes, add observations, and write data stories. Participants can choose from different charts and graphs to analyse the data. Participants can also download Dustbox data in CSV format, as well as save plots and graphs.

## 5. Evaluation

### 5.1. Performance Evaluation with London Air Quality Network (LAQN)

In addition to the Dustbox 2.0 validation, sensor evaluation was performed by comparing the performance with the LAQN New Cross Gate (NXG) monitor. The New Cross Gate area in London is marked by major traffic intersections and key thoroughfares for southeast London. The area includes a mix of housing, cultural spaces, shops and community green spaces. The evaluation campaign aimed to assessing the data quality and address concerns related to the plausibility and consistency of data generated using low-cost sensors. Some of the previous studies [26,48] have highlighted discrepancies in the accuracy of low-cost sensors when evaluated in controlled environments. We performed this evaluation under real-world conditions with varying environmental conditions.

**Figure 13:**
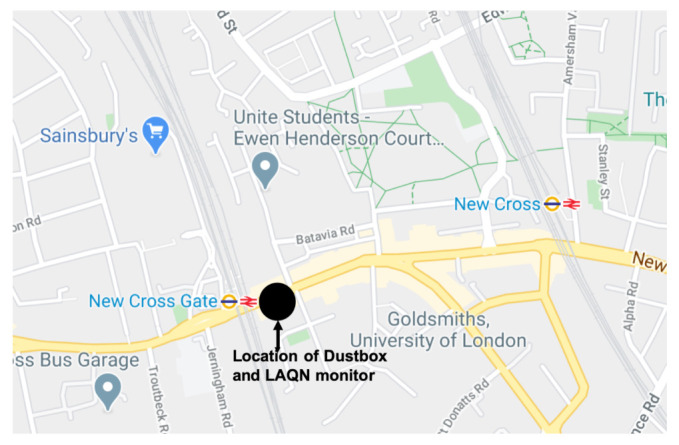
Location of Dustbox 2054 and LAQN monitor.

Dustbox 2054 was collocated with the LAQN NXG monitor. The LAQN monitor is stationed at a roadside location shown in Figure 13 and is positioned 6 m from the road at a height of 3 m. The area is characterised by several busy roads that cross southeast London, as well as a railway with predominantly electric trains, which connects New Cross Gate with central London. Dustbox 2054 was installed on the roof of the monitoring station. It was connected to a power source using a micro-USB cable. The network connection was provided by using a Wi-Fi router placed securely inside the monitoring station. The sampling time was set at one reading every minute.

**Figure 14:**
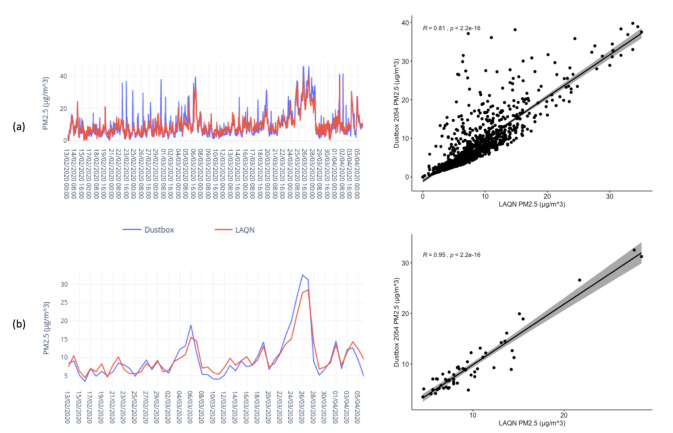
Line plots and scatter plots for Dustbox 2054 and LAQN NXG: (**a**) hly PM2.5 data; and (**b**) daily PM2.5 data.

The colocation experiment was conducted between 13 February 2020 and 5 April 2020. PM2.5 values were averaged over 1 h and 24 h to maintain consistency with the LAQN PM2.5 data. We compared the 1 h and 24 h mean values of PM2.5 levels and found that the Dustbox 2054 showed high correlation, with R values of 0.81 and 0.95, respectively. Figure 14 shows line plots and scatter plots for Dustbox 2054 and LAQN NXG PM2.5 data. It can be observed from Figure 14a that the Dustbox 2054 is able to catch hly variations similar to the LAQN data and shows consistent behaviour with a few outliers. For both 1 h and 24 h mean data, the performance of the Dustbox 2.0 is highly linear with respect to the LAQN PM2.5 data. An important observation was made regarding the capability of Dustbox 2.0 to capture pollution episodes. As observed in Figure 14, there was an increase in daily PM2.5 levels during late March 2020. This was the result of a regional and London-wide pollution incident (https://twitter.com/LondonAir/status/1243165899311497219/photo/1) (accessed on 5 May 2020) between 25 and 27 March 2020. The Dustbox 2.0 and the LAQN monitor show similar patterns, suggesting that the Dustbox 2.0 can successfully capture sudden variations in particulate matter concentration levels in real-world environment.

**Figure 15:**
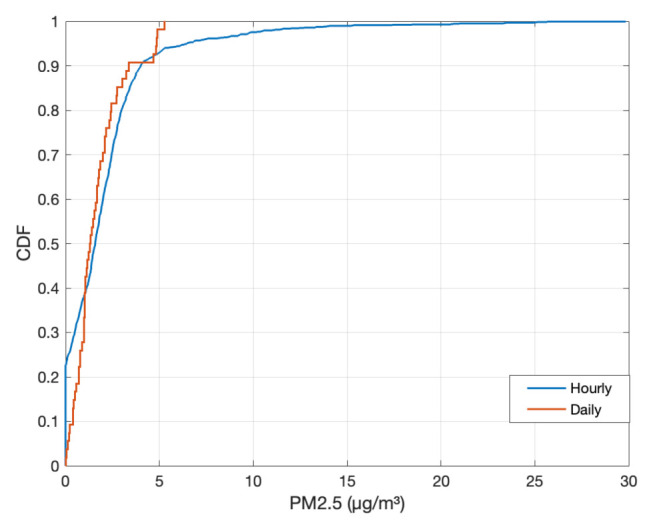
The accuracy of the Dustbox 2.0 compared to that of LAQN monitor.

To further look into the accuracy of Dustbox 2054, we plotted a CDF of accuracy to understand the PM2.5 measurement offset between the Dustbox 2054 and LAQN monitor. As observed in Figure 15, more than 90% of the observations have an offset below 5 μg/m${}^{3}$ for both 1 h and 24 h mean comparisons.

### 5.2. Comparison with State of the Art

We also compared our work with similar state-of-the-art works that monitor PM using low-cost sensors. The works compared here use different technologies and are evaluated under different conditions. Nevertheless, comparing our work with other state-of-the-art works highlights the features and performance of our proposed system. The air quality monitoring frameworks are based on the integration of key building blocks, including the openness of the system, sensor performance, validation, design and scalability of the system. We already highlighted the sensor performance, robust design, data transparency and scalability of the Dustbox 2.0 in previous sections. In Table 5, we compare the features of Dustbox 2.0 with some of the most widely used air quality sensors. In addition to the high accuracy and reliability of data, the Dustbox 2.0 introduces new features in the areas of automatic firmware update as well as robust enclosure design. The Dustbox 2.0 is also part of the AirKit toolkit, which introduces several novel features for analysing and communicating data, providing instructions and documentation, and establishing a citizen-sensing infrastructure as a robust social technology.

Existing air quality monitoring frameworks mentioned in Table 5 use a manual process for updating the firmware. This relies on the user’s resources and ability to perform the update in person. We propose an automatic firmware update procedure that is scalable as well as cost effective. The method is easy to implement and can potentially be used for other IoT frameworks. To ensure the high quality of data, Dustbox 2.0 follows a three-step validation process that includes indoor and field validation with a reference monitor, and colocation experiment with an official monitoring station. In terms of enclosure design, most of the existing works describe a weatherproof enclosure that means the enclosure is water resistant. The Dustbox 2.0 enclosure consists of tiny connected channels on the top that prevents water collection and ensures the suitability of the Dustbox 2.0 for outdoor monitoring even during the rainy season. This was verified during the performance evaluation of the Dustbox 2.0 with the LAQN monitor. During the deployment period, there were thirteen rainy days on an average and two major weather events (Storm Dennis and Storm Jorge) [49].

## 6. Conclusions

The paper presents AirKit, a comprehensive citizen-sensing infrastructure for monitoring air quality. This social IoT-based framework is designed as an open and collaborative system with support from researchers, community members and government agencies. Based on our preliminary findings, we identified the most reliable low-cost sensors and developed two versions of the Dustbox 2.0 device to monitor air quality in a consistent and reliable manner. Using several validation and evaluation methods, it was shown that the Dustbox 2.0 is highly accurate and can record variations and patterns similar to those captured by the reference monitors and the LAQN regulatory monitor in the real-world environment. A viable and secure remote OTA update mechanism is proposed, that is capable of supporting environments having multiple IoT devices. The proposed method automatically updates the firmware every time a new version is available by using an authentication method for remote updates. With respect to existing commercial alternatives, AirKit provides comprehensive documentation and instructions for setting up an air quality study, building and setting up a Dustbox particulate matter sensor, undertaking effective validation and update mechanisms, and using a DIY data-analysis platform for analysing data and proposing actions. Altogether, these integrated components of the AirKit toolkit further the social and environmental potential of IoT technologies.

Our future works are focused on further adding more functionalities to Airkit, including to the Dustbox 2.0, by creating a post-deployment dynamic calibration mechanism; and to Airsift, by enhancing the interoperability of datasets and analysis techniques.

## Figures and Tables

**Table 1:** Comparison between particulate matter sensors.

Device	SidePak	Dustbox 2.0
Manufacturer	TSI	Plantower
Model	AM 520	PMS 5003
Detectable particle size	0.1∼10 microns	0.3∼10 microns
Detectable particle type	PM1, PM2.5, PM5, PM10	PM1, PM2.5, PM10
Operating temperature	0 to 50 (${}^{\circ }$C)	−10 to +60 (${}^{\circ }$C)
Operating humidity	0∼95%	0∼99%
Measurement principle	Light scattering	Laser scattering
	Laser photometer	

**Table 2:** Summary statistics of five Dustboxes at 1 min sampling time.

Device ID	Mean	Median	Variance	Standard Deviation
Dustbox 2048	4.78	3.00	25.99	5.10
Dustbox 2050	5.52	3.00	36.61	6.05
Dustbox 2052	5.72	4.00	38.62	6.21
Dustbox 2061	5.40	3.00	32.05	5.66
Dustbox 2062	5.63	4.00	33.09	5.75
Units for the data: μg/m${}^{3}$				

**Table 3:** Linear regression parameters and measure of linear correlation (R) between PM2.5 concentration (μg/m${}^{3}$) of TSI AM520 and five Dustboxes at 1 min and 1 h time intervals during outdoor colocation.

Device ID	1 min			1 h Average		
	Intercept	Slope	R	Intercept	Slope	R
Dustbox 2048	−1.1	0.6	0.83	−2.3	0.7	0.98
Dustbox 2050	−1.6	0.7	0.85	−2.7	0.8	0.99
Dustbox 2052	−1.4	0.6	0.86	−3.0	0.8	0.99
Dustbox 2061	−1.0	0.6	0.83	−2.4	0.7	0.98
Dustbox 2062	−1.2	0.6	0.80	−2.2	0.7	0.96

**Table 4:** Linear regression parameters and measure of linear correlation (R) between PM2.5 concentration (μg/m${}^{3}$) of TSI AM520 and five Dustboxes at 1 min and 1 h time intervals during indoor colocation.

Device	1 min			1 h Average		
	Intercept	Slope	R	Intercept	Slope	R
Dustbox 2052	−2.0	0.8	0.90	−2.4	0.8	0.99
Dustbox 2053	−4.5	1.0	0.70	−4.9	1.0	0.83
Dustbox 2054	−1.3	0.8	0.64	−2.4	1.0	0.80
Dustbox 2055	−2.6	0.9	0.87	−3.0	0.9	0.99
Dustbox 2059	−2.6	0.8	0.90	−3.0	0.9	0.99

**Table 5:** Comparison with state of the art.

Device	Particles Monitored	Open Source	Validation	Firmware Update	Enclosure Design
Dustbox 2.0	PM1, PM2.5, PM10	Yes	I, F, O	Automatic	Weatherproof, waterproof
Luftdaten [32]	PM2.5, PM10	Yes	F, O	Manual	Weatherproof
SCK 2.1 [19]	PM1, PM2.5, PM10	Yes	F, O	Manual	Weatherproof
PurpleAir [30]	PM1,PM2.5,PM10	No	*	Manual	Weatherproof
RedSpira [50]	PM2.5,PM10	Yes	O	Manual	Partially weatherproof
CanAirIO [38]	PM2.5	Yes	*	Manual	Partially weatherproof
AirBeam2 [31]	PM1, PM2.5, PM10	Yes	I	Manual	Weatherproof

* I: indoor, F: field, O: official monitoring station, *: information not available.

## Data Availability

The data presented in this study are available on request from the corresponding author.
